# Single-sequence based gFET-aptasensors for the discrimination of apo- and holo-RBP4 in human serum

**DOI:** 10.1038/s41598-026-62460-z

**Published:** 2026-07-16

**Authors:** Hu Xing, Runliu Li, Yiting Zhang, Soumya Rajpal, Grigory Bolotnikov, Daniel Gruber, Hans-Maximilian Ruzicka, Jakob Andersson, Anil Bozdogan, Anna Nele Herdina, Robert Strassl, Roger Hasler, Christoph Kleber, Wolfgang Knoll, Boris Mizaikoff, Ann-Kathrin Kissmann, Frank Rosenau

**Affiliations:** 1 Institute of Pharmaceutical Biotechnology, Ulm University, Albert-Einstein-Allee 11, 89081 Ulm, Germany; 2 Institute of Analytical and Bioanalytical Chemistry (IABC), Ulm University, Albert-Einstein-Allee 11, 89081 Ulm, Germany; 3 AIT Austrian Institute of Technology GmbH, Giefinggasse 4, 1210 Vienna, Austria; 4 Division of Clinical Virology, Medical University of Vienna, Spitalgasse 23, 1090 Vienna, Austria; 5 Faculty of Medicine and Dentistry, Danube Private University, Steiner Landstraße 124, 3500 Krems an Der Donau, Austria; 6 Hahn-Schickard, Sedanstraße 14, 89077 Ulm, Germany

**Keywords:** Aptasensor, gFET, RBP4, Molecular dynamics, Molecular docking, Biochemistry, Biological techniques, Biotechnology, Computational biology and bioinformatics

## Abstract

Retinol-binding protein 4 (RBP4) is a key transporter of all-*trans*-retinol (vitamin A), circulating in blood as either holo-RBP4 (retinol-bound) or apo-RBP4 (retinol-free). Dysregulated RBP4 levels, particularly an imbalance between apo- and holo-RBP4, have been implicated in a range of metabolic and cardiovascular diseases. Current detection methods such as ELISA and Western blot lack the specificity to distinguish these two forms. Here, we present an aptamer-based biosensing strategy that overcomes this limitation by using selected single aptamer sequences for the precise and selective quantification of apo- and holo-RBP4 on graphene field-effect transistor (gFET) sensors. The specific contribution to this study is the identification of individual apo- and holo-RBP4-binding aptamers from previously enriched polyclonal libraries, their computational evaluation by molecular docking and molecular dynamics simulations, as well as their implementation as isoform-selective recognition elements on gFET sensors. The polyclonal aptamer libraries previously were generated using FluMag-SELEX (FluMag- **S**ystematic **E**volution of **L**igands by **Ex**ponential Enrichment) that are specific to each RBP4 conformer. Next-generation sequencing and bioinformatic enrichment analyses identified two highly specific and high-affinity aptamers, which were further characterized via computational modelling, including molecular docking and molecular dynamics simulations. This structural approach elucidated the conformer-specific binding mechanisms, demonstrating aptamers’ capacity to differentiate subtle protein conformational changes. The selected aptamers were immobilized on gFET devices, enabling label-free, real-time detection with picomolar sensitivity—a 100-fold improvement over the original libraries. Increased aptamer density on the sensor surface further enhanced signal sensitivity by an order of magnitude. These findings validate the use of aptamers as high-performance biorecognition elements in biosensors, offering a path toward precise RBP4 isoform monitoring.

**Supplementary Information:**

The online version contains supplementary material available at 10.1038/s41598-026-62460-z.

## Introduction

**Fig. 1:**
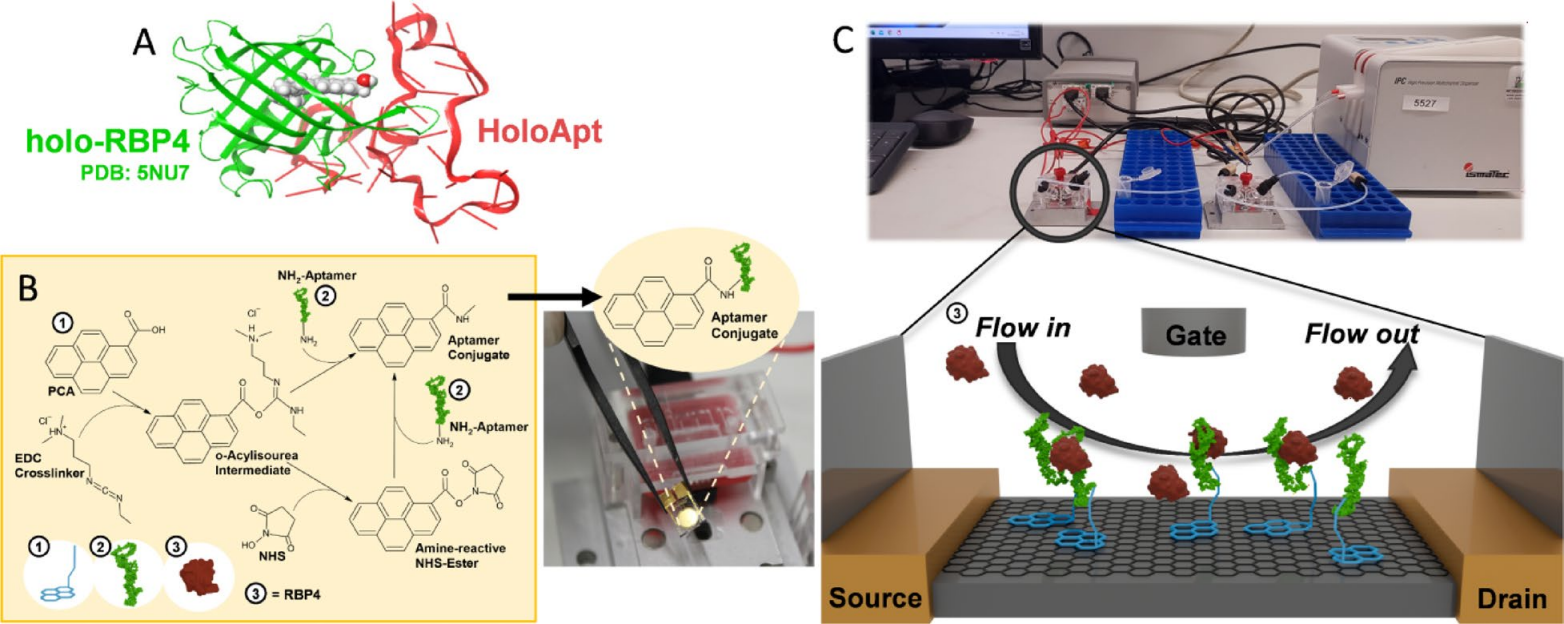
(**A**) Specific aptamer binding to a distinct isoform of RBP4 exemplified by the HoloApt aptamer (red) sequence binding to holo-RBP4 (green) as derived from molecular dynamic simulations. (**B**) Aptamer selection via SELEX and their subsequent use as binding entities on gFET sensor chips. To acquire aptamers specific to either apo- or holo-RBP4 a SELEX is performed starting with ~ 6 × 10^14^ unique sequences (initial library). After multiple rounds of selection, the resulting aptamer pools are sequenced with NGS, and the most promising candidate aptamers are selected through bioinformatical methods. The so found aptamers are then chemically synthesized and used on gFET sensors for further analysis (**C**) Typical gFET measurement setup in a laboratory environment (top) consisting of two flow chambers, a peristaltic pump and source measurement unit. The bottom shows an artistic interpretation of the transistor on the molecular level. The previously synthesized aptamers are chemically linked to pyrene linkers which attach to the reduced graphene layer via π-π stacking. For analysis a constant stream of RBP4 is supplied and the I_DS_ measured at the gate electrode.

Retinol-binding protein 4 (RBP4) is a member of the lipocalin family of ligand-binding proteins^1^, primarily responsible for the transport of all-*trans*-retinol (vitamin A) from hepatic stores to peripheral tissues^2^. To enhance its stability and minimize renal excretion via glomerular filtration, RBP4 bound to retinol (holo-RBP4) forms a complex with transthyretin (TTR)^3,4^. Under physiological conditions, the serum concentration of RBP4 and retinol homeostasis is tightly regulated, ranging from 40 to 60 µg mL^−1^^5^ (2000 to 3000 nM)^6,7^. Altered RBP4 homeostasis has been associated with pathological states such as insulin resistance^8^, type 2 diabetes mellitus^9^, obesity^10^, chronic kidney disease^11,12^, and cardiovascular diseases^13^. It was reported that elevated circulating RBP4 levels in insulin-resistant states, while another study found that the RBP4-to-retinol ratio, rather than total RBP4 alone, is altered in type 2 diabetes^9,14^. In addition, a disbalance of the respective concentrations of both RBP4 forms (Fig. 1A) appears to be crucial for the development of health problems, rather than the pure elevation of protein concentrations, with apo-RBP4 (i.e. the empty version without retinol; Fig. 1A left) being the relevant key marker^14,15^ compared to holo-RBP4 (with bound retinol; Fig. 1A right). The structural distinction between apo- and holo-RBP4 is subtle because both forms retain the overall lipocalin beta-barrel fold^16^. Retinol binding mainly changes the local environment of the hydrophobic ligand-binding pocket and can shift the position or dynamics of pocket-adjacent loops and residues at the barrel opening^17^. These modest local changes are nevertheless relevant for molecular recognition, since aptamers can contact surface-exposed residues and conformationally flexible regions rather than only the buried ligand itself^18,19^. Therefore, discrimination by the selected aptamers is interpreted as recognition of ligand-induced differences in surface topology and dynamics, not as recognition of two globally different protein folds.

Measuring RBP4 levels in blood is currently limited to antibody-based assays including ELISA^20–22^ and a quantitative variant of the traditional Western blotting technique, which has been suggested to be superior for high concentrations of RBP4^20^. Another limitation lies in the fact that the current portfolio of available anti-RBP4 antibodies does not offer the opportunity to differentiate between apo- and holo-RBP4^20–24^. To circumvent this limitation, we have previously developed so called polyclonal aptamer libraries in two independent FluMag-SELEX processes^25^ against RBP4, which were then characterized to specifically bind to apo-RBP4 but not holo-RBP4 and vice versa*.* These polyclonal aptamer libraries suited as specificity-determining binding molecules on electronic sensor chips, allowing precise quantification and discrimination of both RBP4 variants in blood serum with affinities in the low nanomolar range^25^.

Aptamers are an emerging class of short DNA or RNA oligonucleotides with fascinatingly broad applicability in biochemical research and medical diagnostics. Aptamers can adopt specific secondary and tertiary structures, function as binding molecules with specificity and affinity, comparable to those of antibodies^26–28^. Additionally, aptamers offer advantages such as high chemical and physical stability as well as the possibility to easily introduce (chemical) modifications of the molecule^29^. As a proof-of-concept and to demonstrate the principal functionality of the polyclonal libraries to discriminate apo- and holo-RBP4, they were used as binding entities determining specificity on gFET-based electronic sensors and allowed to measure the dedicated protein concentrations in a flow chamber system in a laboratory experimental set-up^25,30^. In this set-up rGO-FETs are functionalized in a cascade of reactions with aptamers (Fig. 1B), which are then used in flow-chambers for the kinetically defined exposure with the respective analyte (Fig. 1C) EG-FETs have emerged as a powerful platform for biosensing applications, offering high sensitivity, label-free detection, and potential for miniaturization and point-of-care diagnostics. They operate on the principle of modulating channel conductivity through the application of a gate voltage, similar to metal oxide FETs. To achieve this, the source and drain electrodes are separated by a semiconductor channel with a gate electrode (Ag/AgCl). Changing the gate voltage modulates the conductivity of the channel leading to an altered current. Similarly, binding of biomolecules alters the local electrostatic environment, inducing a change in the charge carrier distribution, leading to a change in source-drain current (I_DS_)^31^. Observing the I_DS_, while having a constant gate voltage, allows for detection of binding events. Recent studies have demonstrated their versatility especially in combination with aptamers as the binding entities for the detection of a range of analytes from small molecules via proteins to whole bacterial cells^25,30,32–35^. Graphene-based electronic biosensors have also been reported in capacitive sensing formats. It was demonstrated that a graphene oxide-modified double-interdigitated capacitive chip was able to detect SARS-CoV-2 spike protein using EDC/NHS-mediated biomolecule immobilization^36^. This supports the broader relevance of graphene-derived interfaces for label-free electronic biosensing, while the present study focuses specifically on gFET-based discrimination of apo- and holo-RBP4.

In this study, we show the results of the comparative next generation sequencing of the polyclonal anti apo- and anti-holo-RBP4 libraries and the bioinformatic selection of two best performing aptamers, which were highly enriched in their respective library and simultaneously were found to be exclusive for their dedicated target protein. Additionally, in a reverse-engineering strategy, we employed experimentally validated apo- and holo-specific aptamers to computationally model their interactions with the respective conformers of RBP4 and to elucidate the molecular basis of their target specificity. Given the subtle structural differences between the apo- and holo-forms, such modelling enables a mechanistic understanding of how aptamers achieve conformer specificity—a capability rarely observed even in conventional antibodies. To this end, we established a structure-guided computational workflow comprising a sequence of secondary structure prediction, 3D-models generation, molecular docking and molecular dynamics simulations. This approach can enable the identification of critical contact residues and conformational motifs responsible for binding affinity and selectivity. The high conformational plasticity of aptamers both in their unbound state and upon target engagement—poses unique modelling challenges not typically encountered with small molecules or rigid ligands^37,38^. Addressing these complexities via computational design can reduce experimental screening efforts and accelerate the rational engineering of high-performance aptasensors. This study underscores the transformative yet underutilized potential of Computer-Aided Aptamer Design (CAAD) in precision diagnostics, especially for low-abundance or conformationally dynamic protein biomarkers. The identified aptamers were then implemented to construct apo- and holo-RBP4 specific gFET aptasensors, which were subsequently characterized. The detection was specific and represented, with affinities of approximately 50 picomolar, a great improvement compared to the polyclonal aptamer libraries. Moreover, in combination with the improved density of specific aptamers on the chip electrode surface the sensitivity was increased 1000-fold. We believe that the selected individual aptamers and their performance in the initial experiments using the laboratory setup will open new avenues to develop not only gFET based aptasensors for the routine quantification of potential RBP4 disbalances, but may also inspire the community to drive the aptasensor technology per se to perfection, including the development of multiplexed sensors for point-of-care devices.

## Materials and methods

### Protein production, refolding and retinol loading

Apo- and holo-RBP4 have been recombinantly produced in commercial *Escherichia coli* BL21 DE3 Tuner expression strains, refolded and loaded with retinol as described earlier^25^.

### Illumina sequencing of aptamer libraries and bioinformatical analysis of representative single aptamers

The aptamer libraries apo-R4, apo-R8, holo-R4 and holo-R8 were sent to Eurofins Genomics (Konstanz, Germany) for NGS Illumina amplicon sequencing. For this purpose, the target library needs to be PCR amplified twice to introduce the adapt and index sequences required for sequencing. First, in the first round of PCR amplification, the forward primer: 5´-ACGATGATACTCGGACTGTAGGGAAGAGAAG GACATATGAT-3′ and the reverse primer: 5´-TCTCGTGTTCAAGCGACTCAAGTGGT CATGTACTAGTCAA-3′ were used to introduce the universal primer binding site. The amplification reaction was performed using Herculase II Fusion DNA Polymerases (Agilent Technologies, Inc., Santa Clara, California, USA) and was divided into three thermal cycles: 95 °C pre-denaturation for 3 min; 95 °C denaturation for 30 s, 56 °C annealing for 30 s, and 72 °C extension for 10 s, for a total of 9 cycles; and 72 °C final extension for 2 min. The purified PCR product was then used as a template for a second round of PCR to introduce the index sequence for parallel sequencing of the aptamer library. For apo R4, apo R8, holo R4, and holo R8 libraries, primers 5′-TCAGTCGTATATCACGACGATGATACTCGGACTG-3′ and 5′-GCTATGTACTCGT GATTCTCGTAGTTCAAGCGAC-3′ and primers 5′-TCAGTCGTATCGATGTACGATGA TACTCGGACTG-3′ and 5′-GCTATGTACTACATCGTCTCGTAGTTCAAGCGAC-3′ were used for differentiation. NGS data were then quality controlled using FastQC [S. Andrews, Fastqc: A Quality Control Tool for High Throughput Sequence Data., http://www.bioinformatics.babraham.ac.uk/projects/fastqc., accessed: 11.06.2025], sequences were sequenced, adapter sequences were trimmed using the FASTX toolkit, and nucleotide distribution analysis was performed. Finally, the secondary structure of the sequences was predicted using the FASTAptamer toolbox (v1.0.3) [Khalid K. Alam, Jonathan L. Chang & Donald H. Burke “FASTAptamer: A Bioinformatic Toolkit for High-Throughput Sequence Analysis of Combinatorial Selections.” Molecular Therapy—Nucleic Acids. 2015. 4, e230, https://burkelab.missouri.edu/fastaptamer.html, accessed: 11.06.2025]^39^ and the Mfold server (v4.7) [M. Zuker, Nucleic Acids Res 2003, 31, 3406., https://www.unafold.org/mfold/applications/rna-folding-form.php, accessed: 11.06.2025].

### Computational workflow

**Fig. 2:**
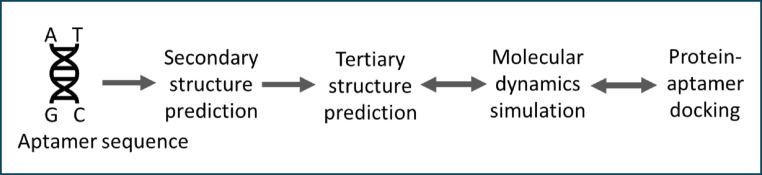
Schematic representation of the workflow for investigating DNA aptamer-protein interactions with apo and holo forms of RBP4. The workflow involves initial prediction and optimization of the DNA aptamer’s secondary (2D) and tertiary (3D) structures, followed by molecular dynamics simulations to refine structural stability. Protein-aptamer docking is then conducted to elucidate binding interactions and analyze the structural basis of aptamer-target recognition.

All computational analyses were conducted using the Schrödinger Suite (v2023-2) [accessed: 11.06.2025, https://www.schrodinger.com/life-science/download/release-notes/release-2024-2/]. Experimentally identified aptamer sequences were first folded into their predicted secondary structures using Mfold (v4.7) [M. Zuker, Nucleic Acids Res 2003, 31, 3406., https://www.unafold.org/mfold/applications/rna-folding-form.php, accessed: 11.06.2025]. The most thermodynamically stable 2D conformations were manually reconstructed as 3D structures using the Biopolymer Builder in Maestro [Schrödinger Release 2026-2: Maestro, Schrödinger, LLC, New York, NY, 2025., https://www.schrodinger.com/life-science/download/release-notes/release-2024-2/, accessed: 10.06.2026]. These 3D models were processed through LigPrep [Schrödinger Release 2026-2: LigPrep, Schrödinger, LLC, New York, NY, 2025., https://www.schrodinger.com/life-science/download/release-notes/release-2024-2/, accessed: 10.06.2025] to assign protonation states (pH 7.4), generate low-energy tautomers, and optimize geometries. Conformational sampling was also performed with ConfGen [Schrödinger Release 2026-2: ConfGen, Schrödinger, LLC, New York, NY, 2025, https://www.schrodinger.com/life-science/download/release-notes/release-2024-2/, accessed: 11.06.2025] (default settings, up to 50 conformers per aptamer), and conformers showing agreement with predicted 2D motifs were selected for docking. The apo and holo forms of human RBP4 were prepared using the Protein Preparation Wizard with default OPLS4 force field settings [Schrödinger Release 2026-2: Force Fields, Schrödinger, LLC, New York, NY, 2025, https://www.schrodinger.com/life-science/download/release-notes/release-2024-2/, accessed: 11.06.2025]^40^. Protein-aptamer interactions were modeled using the protein–protein docking workflow^41,42^ (Fig. 2). Docked complexes were analyzed for binding site preference, hydrogen bonding and interaction energy trends across the apo and holo states.

### Chip functionalization

Commercial Source-Drain substrates (Micrux, L = 5um) with reduced graphene oxide (rGO) as channel material were fabricated as previously described^43^. For functionalization, 500 μM PyPEG (sealing agent polyethylene glycol with PBSE connector pre-fix) and 50 μM 1-pyridine carboxylic acid (PCA) were dissolved in DMSO. The gFETs were immersed in the 1:10 mixture and reacted at room temperature in the dark for 24 h. Subsequently, the gFETs were washed twice with 1 mL of isopropanol and carefully dried under nitrogen. Briefly, the pyrene-containing linker molecules adsorb to graphitic rGO domains via π–π stacking, providing carboxyl groups for subsequent EDC/NHS activation. EDC converts surface carboxyl groups into reactive O-acylisourea intermediates, which are stabilized by NHS to form amine-reactive NHS esters. The 5′-amino-modified aptamers then react with these activated groups to generate covalent amide bonds on the sensor surface. Absolute coupling efficiency and aptamer surface coverage were not directly quantified, but functionalization was instead monitored electrically by I_DS_V_G_ measurements after each modification step and functionally verified by the conformer-specific RBP4 response^25,31,32,44^. To monitor the bio-recognition, the transfer characteristic of the gFET (I_DS_V_G_) was recorded using a Keysight U2722A modular source/measure unit (Keysight Technologies, USA) and a custom developed LabView-based software (National Instruments, USA). A specific range of gate voltages (− 0.5 V to + 0.5 V) at a scan rate of 20 mV/s with V_DS_ = 50 mV was used as system parameters and the I_D_V_G_ curves were recorded. First, the chip surface was rinsed with distilled water at a flow rate of 0.2 mL/min for 10 min, followed by rinsing with 0.01 × DPBS at a flow rate of 0.5 mL/min for 2 min. Afterwards, the I_DS_V_G_ were recorded. Then, for aptamer-coupling 1 mL of fresh 0.01 × DPBS solution containing EDC (15 mM) and NHS (15 mM) was prepared and to the chips were rinsed at a flow rate of 0.2 mL/ min for 30 min to fully activate the carboxyl groups on the chip surface. Then, the system was rinsed with 0.01 × DPBS at a flow rate of 0.5 mL/min for 1 min, followed by reducing the flow rate to 0.2 mL/min and continuing to rinse for 10 min to completely remove any residual EDC and NHS. Then, 1 mL of 1 × DPBS containing 100 pmol of activated amino-labeled single aptamer was circulated at a flow rate to 0.2 mL/min for 1 h, allowing the activated aptamer to fully attach to the chip surface, thereby completing chip functionalization. Again, the I_DS_V_G_ were recorded to verify successful aptamer coupling.

### Aptamer characterization

*Determination of K*_*d*_* values of aptamer-functionalized gFET*. Different concentrations of apo-RBP4 and holo-RBP4 were prepared according to the concentration gradient of 0, 0.03, 0.3, 3, and 30 nM in 1 mL 1 × DPBS. The detection system was set up according to the method in Section "Chip Functionalization”, and the corresponding I_DS_V_G_ curves under different concentration gradients were recorded respectively. After normalization, the Dirac point voltage was evaluated, and the curve of Dirac point voltage change with the concentration of the test substance was fitted by the one site specific binding model of nonlinear regression using GraphPrism 8 software.

*Quantitative tracking of apo-RBP4 and holo-RBP4 in defined mixtures*. Apo-RBP4 and holo-RBP4 were mixed in different proportions to keep the total protein concentration at 0.3 nM in 1 mL 1 × DPBS and the ratios of apo-RBP4 and holo-RBP4 were 0%, 25%, 50%, 75% and 100%, respectively. Subsequently, gFETs functionalized with ApoApt and HoloApt were used to record I_DS_V_G_ curves at the different ratios. The Dirac point voltage was obtained according to the method in 2.3.1, and the trend of the Dirac point voltage changing with the ratio of the concentration of the detected substance was described using Origin software.

*Quantitative tracking of apo-RBP4 and holo-RBP4 in human serum*. To quantify RBP4 in the presence of commercial human serum (Product Number H4522-20ML, Merck KGaA, Darmstadt, Germany), 1 mL of 1% serum sample was prepared with 1 × DPBS, and different concentrations of apo-RBP4 and holo-RBP4 were added, and the concentrations of the two changed according to the gradient of 0, 0.03, 0.3, 3, and 30 nM, respectively. Subsequently, gFETs functionalized with ApoApt and HoloApt were used to record the corresponding I_DS_V_G_ curves under different concentration gradients. After normalization, the ΔI_DS_ change compared to the buffer at -0.5 V was recorded. The commercial serum was used without prior retinol depletion. Therefore, the experiment was designed as a spike-in recovery measurement in a complex serum matrix rather than as proof that endogenous serum RBP4 was exclusively present in the apo form. Defined recombinant apo-RBP4 and holo-RBP4 were added to diluted serum and apo-/holo-RBP4 contributions were assigned based on the known identity of the spiked recombinant proteins and the conformer-specific responses of ApoApt- and HoloApt-functionalized gFETs. Thus, the assay evaluates detection of added apo- and holo-RBP4 in serum but does not independently confirm the retinol-binding state of endogenous serum RBP4. It is This is important to demonstrate that the aptasensors measurements could be performed in a human serum matrix using defined recombinant apo- or holo-RBP4 spike-ins.All concentration-dependent measurements were performed using three independently functionalized gFET chips measured with the same electronic measurement setup. Data are present as mean ± standard deviation. Thus, the error bars reflect chip-to-chip variation under identical measurement conditions.

## Results

**Fig. 3:**
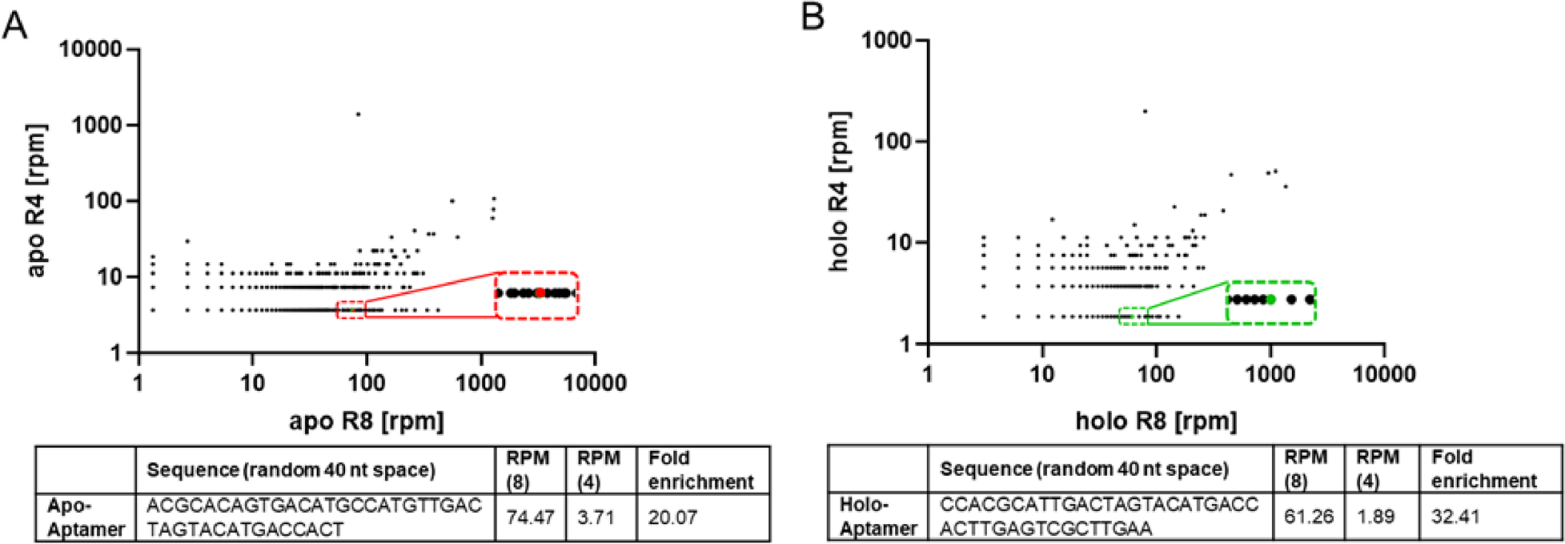
Bioinformatical analysis of the apo- and holo-aptamer libraries sequences. Scatter plots of sequence abundances from NGS sequencing of final rounds 8 and early rounds 4 for (**A**) apo-RBP4 and (**B**) holo-RBP4 with most enriched and exclusive selected sequences.

The use of polyclonal (i.e. focused) aptamer libraries is in most cases already sufficient to allow precise measurements in sensing applications^30,45–52^ including the construction of aptamer functionalized gFET sensors^25,30,34,35^. However, the ease to produce individual aptamers chemically as compared to enzymatic amplification of a library, qualifies the use of selected individual aptamers as the preferable option for the development of sensor devices. Such sequences are available in the already characterized library as the essentially functional part of the sequence space. The polyclonal anti apo-RBP4 and anti holo-RBP4 rounds four (early) and rounds eight (final) libraries^25^ were subjected to next generation sequencing (NGS) and subsequent bioinformatic analysis as described earlier^34,51,53^. The aptamers ApoApt and HoloApt were identified as the most enriched and library-exclusive candidate sequences in the apo- and holo-RBP4 selections (Fig. 3A, B). This selection step provided the sequence-defined recognition elements used for the subsequent computational and gFET-based characterization.

**Fig. 4:**
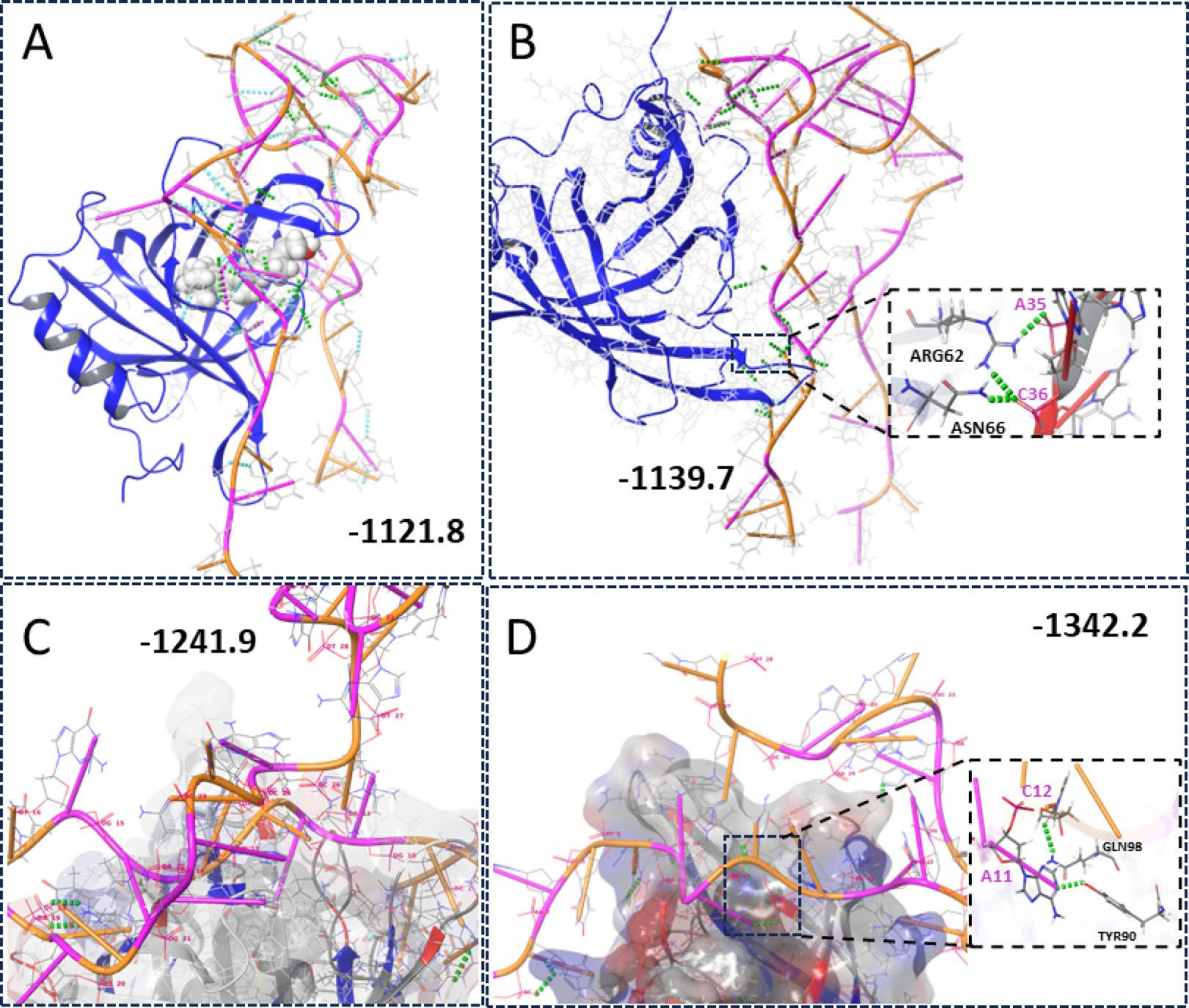
Comparison of binding interactions for selected aptamers with apo and holo forms of the RBP. (**A**, **B**): Binding conformations of an apo-specific aptamer with holo and apo form of RBP, respectively. (**C**, **D**): Binding sites and hydrogen bonding differences for a holo-specific aptamer with apo and holo form of RBP, respectively. Estimated binding energy [kcal/mol] is showed in each case where higher negative energy scores indicate higher binding affinity.

Next, the *in-silico* performance of the selected sequences of ApoApt and HoloApt with apo- and holo-RBP4 was evaluated (Fig. 4) using a structure-guided computational workflow (Fig. 2). Aptamers are highly specific, sequence-defined biorecognition elements capable of forming intricate nucleic acid amino acid interaction networks with target proteins. While DNA aptamers offer greater chemical stability than their RNA counterparts, their structure–function relationships remain underexplored due to limited structural data and high conformational plasticity. To address the challenge of decoding aptamer-target interactions, we implemented a computational framework not focused on generating a definitive structure, but on mapping plausible interaction motifs for rational design—ultimately surpassing the limitations of iterative SELEX procedures. The first step derives secondary structures using Mfold to identify thermodynamically favourable folding patterns of the core aptamer regions (Fig. S1). These were then translated into 3D structures using Maestro’s Biopolymer Builder. LigPrep and ConfGen were subsequently employed to generate a diverse pool of conformers, capturing a range of folding topologies based on the predicted 2D architectures. Multiple low-energy conformers were obtained, each differing subtly in spatial arrangement and potential interaction sites. This structural multiplicity, while biologically realistic, complicates the identification of a single “accurate” 3D model. Further, each conformation—when subjected to MD optimization and protein docking—adopts new energetically favourable poses upon interaction with the target protein. These observations form the basis to shift from a structure-centric paradigm to an interaction-centric analysis. Instead of relying solely on structural accuracy, our approach emphasizes the diversity and recurrence of interaction motifs, offering mechanistic insights into aptamer specificity despite conformational ambiguity. Then, conformers exhibiting consistent secondary and tertiary motifs were selected for further analysis. At each modeling stage, 50 conformers were generated using ConfGen, and initially top 10 structures were shortlisted both low-energy profiles and structural fidelity to the predicted 2D motifs. These were subsequently docked with the apo and holo forms of RBP4 to assess interaction consistency across the conformational ensemble. For each docking run, the top 10 binding poses (ranked by PIPER pose energy) were examined to extract recurring interaction patterns and key amino acid residues involved. This ensemble-based approach focuses on extracting consistent mechanistic patterns rather than relying on a single conformer. While a full-scale per-residue energy decomposition of the > 400 complexes generated will be reported separately, we highlight here recurring residue-level interactions that appeared across top-ranked conformers. One of the top-ranked structures revealed target specificity: apo-specific aptamers displayed stronger interactions with the apo-RBP4 form; in parallel, the holo pair showed an even larger differential in binding energies and conformational complementarity. The holo-specific aptamer displayed a docking score of − 1342.2 kcal/mol with holo-RBP4 versus − 1241.9 kcal/mol with the apo form, suggesting preferential binding in the convergent pair, which is consistent with experimental binding shifts (Fig. 4). Importantly, the reported docking scores represent PIPER pose energies; these are not absolute free energies but composite scoring terms that often reach large magnitudes for extended interfaces such as aptamer–protein complexes. As such, these values are interpreted as relative comparisons among apo versus holo complexes and across aptamer conformers. Further validation via molecular dynamics simulations and more rigorous per-residue energy decomposition will confirm which contacts consistently contribute to binding specificity^54^. The fingerprint analysis (Fig. 2S) also substantiates the differential binding observed in the docking scores. The holo-specific aptamer displayed a distinct interaction profile, with enriched contacts at unique sites in the holo form, particularly among residues ARG121, ASP131, TYR133 (Fig. 2S, A). Conversely, for the apo-specific aptamer, only minor differences were observed between the apo and holo forms, particularly in residues starting from ARG163, which is also reflected in the docking scores, showing only slight variations, nevertheless, supporting the finding of specificity i.e. not binding the holo form (Fig. 2S, D).

**Fig. 5:**
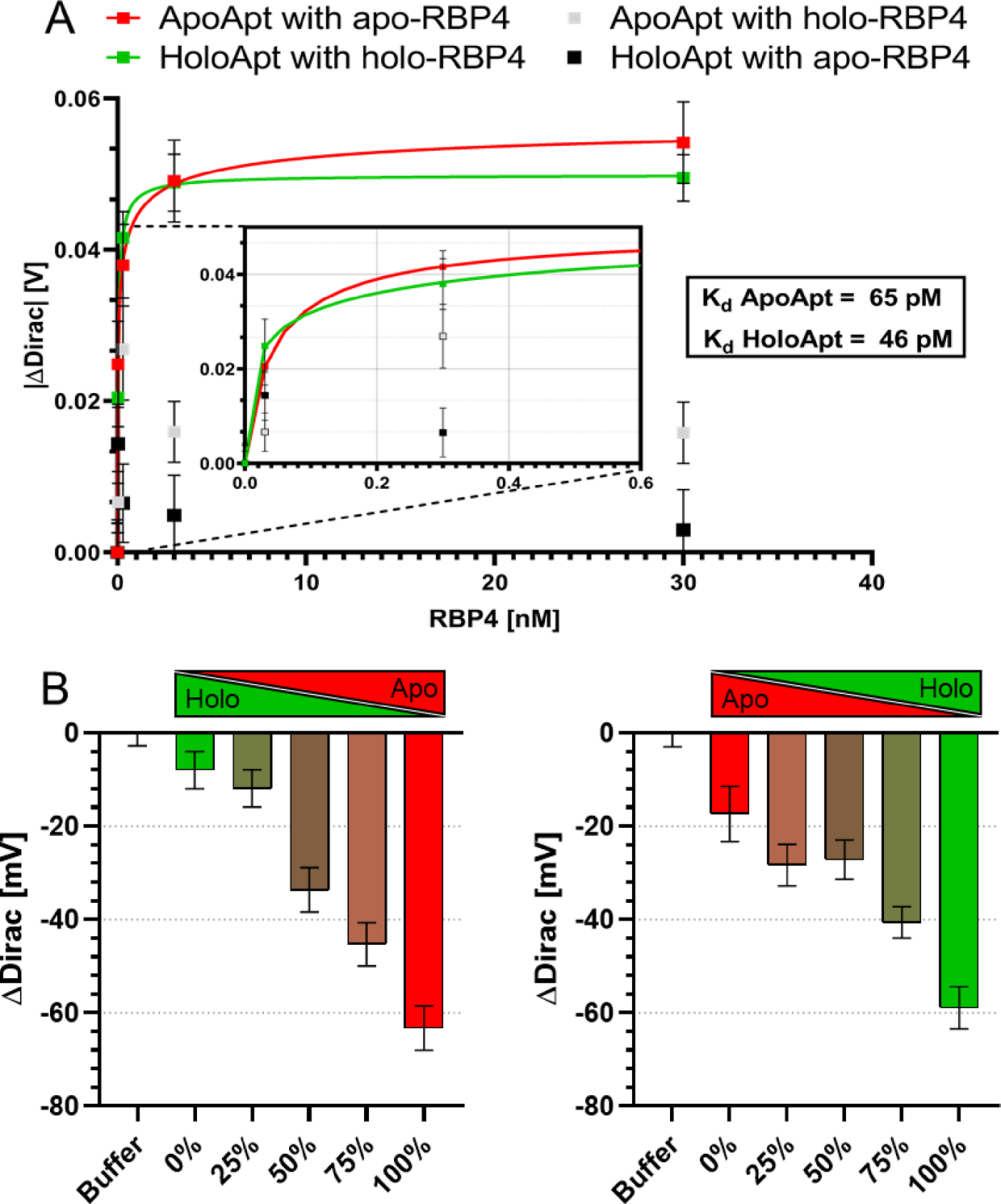
Specificity ApoApt (red) and HoloApt (green) derived from I_DS_V_G_ measurements shown in Fig. S4 used to derived (**A**) K_d_ values of ApoApt K_d_ = 65 pM and HoloApt K_d_ = 46 pM. (**B**) Specificity shown in defined mixtures (0–100%) of apo- (red) and holo-RBP4 (green). Error bars indicate standard deviations derived from three independently functionalized gFET chips measured under identical conditions.

The computationally supported candidate aptamers were then tested experimentally on rGO-FET sensors to evaluate apparent affinity and isoform discrimination (Figs. 5; S4). The first results established a robust framework for reverse-engineered modeling, providing a computational approach for tracing the interaction pathways that govern aptamer specificity. Moving beyond post hoc validation, this methodology enables the transition towards predictive design, facilitating the integration of more advanced mechanistic modeling techniques such as but not limited to per-residue interaction energy decomposition, molecular dynamics simulations, and the potential incorporation of machine learning. These will in turn enhance the precision and predictability of aptamer binding profiles for ultimately advancing the design of highly selective aptamers for therapeutic and diagnostic applications. ApoApt and HoloApt were chemically synthesized as amino-group labeled variants (5′) and used to functionalize reduced graphene oxide modified gFETs (rGO-FETs) to equip them with the specificity determining aptamers as the functional binding entities. The presence of the aptamer functionality on the rGO-FET was evaluated upon coupling with EDC-NHS (Fig. 1B) as described previously for proteins^25,44^ and cells^34^ and implementing the established gate-voltage (V_G_) scanning protocol from − 0.5 to + 0.5 V^25^. The commercial chips were initially coated with graphene, subsequently chemically reduced by hydrazine treatment followed by coating with PyPEG providing accessible carboxy-groups. The sequential functionalization of the surfaces of each chip used with amine-labeled aptamers upon EDC/NHS activation (“layer-by-layer” functionalization) was characterized by I_DS_V_G_ evaluation prior to the respective individual quantitative sensor measurements (Fig. S3). The aptamer-modified chips were able to capture the dedicated target proteins present in the samples as expected, as this resulted in the induction of distinct shifts in the I_DS_V_G_ curves by altering the charge distribution within the semiconductor structure. Recording the I_DS_V_G_ curves of samples containing increasing concentrations of either apo- or holo-RBP4 (0–30 nM) revealed increased shift alterations (ΔI_DS_) depending on the increase in the protein amount measured at a constant V_G_ at − 0.25 V (Fig. S4). Specificity was determined by a similar set of experiments using either the concordant protein-aptamer pairs (i.e. ApoApt-apo-RBP4 and HoloApt-holo-RBP4) or the respective divergent pairs (i.e. Apo-Apt-holo-RBP4 and HoloApt-apo-RBP4) with the latter only delivering marginal background signals (Fig. S4). These measurements allowed the calculation of dissociation constants (K_d_) for the concordant specific pairs of analyte and aptamer of 65 and 46 pM (ApoApt < HoloApt) (Fig. 4A) and thus representing an at least tenfold improvement as compared to the original polyclonal libraries^25^, respectively. As expected for the specific binding of the selected individual aptamers the control measurements using the respective control isomer completely failed in delivering K_d_ values (Figs. S4A and 5A). For the intended application to discriminate apo- from holo-RBP4 it was necessary to show that quantitative measurements of each isomer were possible in the presence of the other isomer as a “contamination”. In mixtures of both proteins with increasing concentrations of one isomer with a fixed final total concentration of 3 nM RBP4 (0, 25, 50, 75 and 100% of the respective isoform) the concentration gradient could easily be followed (Figs. S4B and 5B). The apparent difference between affinity and specificity should be interpreted with caution because K_d_ and conformer discrimination describe distinct properties. The fitted K_d_ values reflect the apparent affinity of the immobilized aptamers toward their cognate purified targets under the selected fitting conditions, whereas specificity in the mixture experiment depends on the ratio of cognate response to non-cognate cross-response. Thus, the lower K_d_ of HoloApt indicates stronger apparent binding to holo-RBP4 but does not necessarily imply superior discrimination between holo- and apo-RBP4. ApoApt may display higher apparent specificity because its cross-response toward holo-RBP4 is lower, whereas HoloApt may retain partial response toward apo-RBP4 due to the close structural similarity of the two RBP4 conformers. Since apo- and holo-RBP4 share the same overall lipocalin fold and differ mainly in local ligand-induced surface topology and dynamics, partial overlap of aptamer-accessible epitopes is mechanistically plausible.

**Fig. 6:**
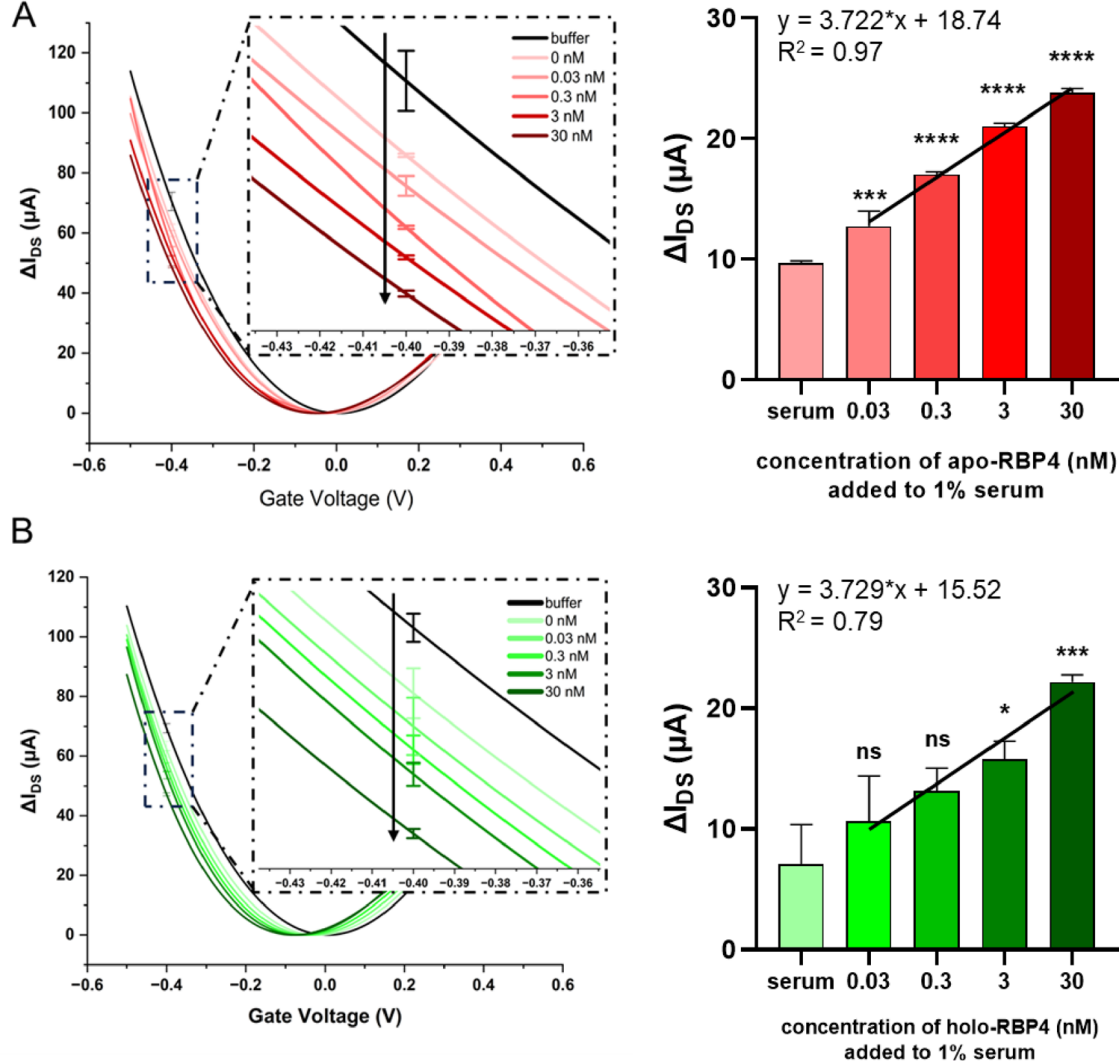
Validation of functionality using 1% human serum spiked with increasing concentrations of (**A**) apo- (red) or (**B**) holo-RBP4 (green) (0.03–30 nM). I_DS_V_G_ characterization of sensing devices obtained using sweeping the gate voltage from − 0.5 to 0.5 V. I_DS_V_G_ curves represent mean values of experiments conducted in triplicate. The bar diagrams (right sides) shown the ∆I_DS_ at -0.5 V gate voltage as measured in the original curves (left sides). Error bars indicate standard deviations from three independently functionalized gFET chips measured under identical conditions. Statistical significance was assessed by one-way ANOVA followed by Dunnett’s multiple comparisons test versus the serum reference. Significance levels are indicated as as *p* < 0.05, **p < 0.01, *p < 0.001, ***p < 0.0001; n.s., not significant.

After confirming buffer-based affinity and isoform discrimination, the aptamer-functionalized sensors were tested in diluted human serum to evaluate whether target-dependent responses could still be resolved in a complex matrix (Figs. 6 and S5). It is important for an intended application to demonstrate that the aptasensor measurements could be performed with RBP4 being present in human blood serum. It was previously observed that the viscosity of pure serum was too high for measurements in the flow chambers, but dilutions to 10% (*v/v*) or lower to 1% (*v/v*) were perfectly suited in this experimental set-up^25^. To meet application relevant (serum) concentrations of RBP4 (apo- and/or holo-) dilution series ranging from 0.03 to 30 nM were prepared by spiking serum with the proteins added in PBS buffer and were subsequently measured. The increase of signals along these dilutions was clear and correlated linearly from the lower border 0.03 nM to the highest concentration of 30 nM (Fig. 6), which was also observed similarly in the Dirac points for both measurements (Fig. S5). The increasing ∆I_DS_ values upon addition of defined target concentrations show that additional RBP4 can be resolved in diluted serum. For ApoApt-functionalized sensors, a significant difference from unspiked serum was already observed after addition of 0.03 nM apo-RBP4 (linear dynamic range: 0.03 nM to 30 nM), whereas HoloApt-functionalized sensors showed a significant difference from 3 nM holo-RBP4 (linear dynamic range: 3 nM to 30 nM) onward. Thus, the data demonstrate proof-of-principle matrix compatibility and target detection in the tested low-nanomolar range. To avoid ambiguity between binding affinity and analytical sensitivity, the K_d_ values and the concentration range of the gFET response are reported separately. The apparent K_d_ values were obtained from nonlinear fitting of the concentration-dependent response in buffer, whereas the analytical working range in serum was defined from the spike-in calibration shown in Fig. 6. Thus, the data defines the concentration intervals that were experimentally resolved under the present assay conditions: 0.03–30 nM for ApoApt and 3–30 nM for HoloApt. These intervals should be interpreted as assay-specific working ranges rather than formal analytical detection limits.

## Discussion

The concentration of RBP4 in serum of healthy individuals is in the range of 2000 to 3000 nM^6,7,55^. Erikstrup et al. reported that RBP4 levels appear to be reduced in patients with type-2-diabetes and/or adipositas^14^ with the ratio of RBP4-to-retinol and thus apo-RBP4 to holo-RBP4 being elevated and is therefore crucial and decisive to determine the health status of candidate patients. Thus, the disease-relevant parameter may not be total RBP4 alone, but rather altered RBP4-retinol homeostasis. Since the RBP4-to-retinol ratio may indirectly reflect changes in the relative abundance of retinol-bound holo-RBP4 and retinol-free apo-RBP4, methods capable of discriminating both RBP4 forms are needed for a more detailed assessment of RBP4-related disease states. Therefore, improved sensitivity is important not because native serum RBP4 is expected to be present only at low concentrations, but because serum samples often require dilution to reduce matrix effects and to bring the analyte into the calibrated working range of the sensor. High sensitivity also allows changes in apo- or holo-RBP4 levels to be resolved after dilution, which is relevant when changes in the RBP4-retinol balance rather than total RBP4 alone are of interest. Plasmon resonance-based sensors for the sum-detection (apo- plus holo-RBP4) without quantitative discrimination of both isoforms have been described based on an aptamer with considerably unpropitious affinity and resulting low sensitivity^56^. We previously reported a gFET-based electronic sensor with comparably higher and sufficient sensitivity, which allowed quantitative measurements of RBP4 concentrations as low as 300 nM in diluted human serum (10% (*v/v*)) and was equipped with a so-called focused aptamer library against apo- or holo-RBP4 as the specificity-determining molecular entity^25^. To gain additional technical range and flexibility to use higher dilutions of serum from patient samples for future applications, it is advantageous to increase sensitivity while maintaining the sufficient specificity. A generally reasonable approach is to increase the density of specific binding molecules on the sensor surface, which can easily be achieved by replacing the polyclonal mixture of aptamers residing in the library by isolating individual aptamers. We therefore used NGS in combination with the established bioinformatic analyses to compare the respective SELEX rounds 4 with the final round 8 and finally selected the two most enriched aptamers present in the apo- and holo-RBP4 libraries. Subsequently, these sequences were checked for exclusiveness (i.e. presence in one library but completely absent in the other). The resulting two aptamer molecules ApoApt and HoloApt used to functionalize rGO-FETs allowed specific and sensitive quantification of the RBP4 isoforms and discrimination of apo- from holo-RBP4, respectively. Measurements could be made without difficulty in human serum diluted to 1% (*v/v*) and provided a sensitivity low as 0.03 nM, which is 1000-fold below the highest normal serum levels and low enough for all expectable demands in RBP4 diagnostics. The serum experiments were designed as spiking experiments to test whether the aptasensors remain functional in a complex matrix rather than to establish final clinical decision limits. A 100-fold dilution of physiological RBP4 concentrations corresponds to approximately 20–30 nM and the response in this upper range is less pronounced than at lower concentrations. However, the detection of significant signal changes after addition of 0.03 nM apo-RBP4 and 3 nM holo-RBP4 to diluted serum shows that additional target protein can be detected despite the matrix background. This supports the applicability of the sensor principle in serum, while future validation with native clinical samples the final clinically relevant resolution. Although HoloApt showed a slightly lower K_d_ than ApoApt in buffer, this was not directly reflected in the serum-based gFET response. This apparent discrepancy exists for example through the fact that K_d_ reflects binding affinity, whereas the gFET signal depends on additaional parameters, including aptamer orientation on the sensor surface, distance of the bound protein from the graphene channel, charge redistribution upon binding and matrix effects from serum. These high-affinity aptamers may reduce sample volume, because only a small amount of blood is needed into the calibrated sensor range. This may be advantageous for future applications with patients or studies where sample availability is limited. Moreover, this represents in sensitivity of three orders of magnitude compared to the original library-based sensors^25^. The polyclonal or “focused” libraries have been shown to potentially outperform individual aptamers in the precise and robust detection of a broad target spectrum (e.g. different clinical isolates of the same pathogenic bacterial species)^53^. However, the use of aptamer libraries in technical devices or diagnostic techniques then requires a (PCR-based) production technology to synthesize the respective mixture of productive individual aptamer sequences (the complete sequence space of the functional library). Although this has been shown to be possible with reasonable reproducibility over generations of library production processes, the use of individual aptamers selected from the libraries offer an essential advantage for the development of diagnostic tools, since they can be synthesized chemically and thus highly reproducibly. In combination with the possibility to increase sensitivity as demonstrated in this study this concept to select individual aptamers by the combination of NGS and bioinformatic analyses involving comparison of early and late SELEX rounds with subsequent isolation of library exclusive molecules represents a straightforward way to base sensor devices on the best-performing binding entities.

The biological function of aptamers is not encoded in a single static structure, but in a dynamic ensemble of conformations it can adapt upon binding. The ability of distinct conformers to exhibit comparable binding energies suggests a landscape of functionally competent structures rather than a single optimized fold. By focusing on recurring interaction motifs across multiple conformers, the CAAD framework presented in this study acknowledges the inherent structural plasticity of aptamers. In fact, such flexibility may be key to the ability of aptamers to recognize diverse states of a target protein, such as its apo and holo forms, as demonstrated in the docking results. Absolute docking energies provide a useful baseline, but patterns of relative differences between apo vs. holo complexes, together with MD-based refinement, generate robust evidence of binding specificity. Ultimately, CAAD holds the potential to redefine aptamer discovery through intelligent pre-screening of sequence libraries, rapid adaptation to mutational variants, and prediction of binding specificity across protein isoforms or post-translationally modified targets. With aptamers uniquely capable of sensing subtle conformational variations, CAAD emerges as an essential tool to design molecular probes for dynamic biomarkers, such as cleaved, phosphorylated, or allosterically regulated proteins. Furthermore, CAAD can facilitate the design of switchable aptamers, drug-conjugated therapeutic constructs, and cooperative multi-epitope binders. The computational workflow provides important mechanistic insight into the experimentally observed conformer specificity of the selected aptamers. Rather than relying only on sensor output, the CAAD approach helps identify plausible aptamer-RBP4 interaction motifs and local contact regions that may contribute to apo-/holo-RBP4 discrimination. The docking scores were interpreted as relative indicators of interaction trends within the same computational workflow, not as absolute binding free energy. This is important because aptamer-protein complexes are conformationally flexible and docking outcomes depend on the selected aptamer conformers and scoring functions. The experimentally observed gFET responses support the predicted conformer preference at a functional level, while high-resolution structural studies would be required to define one unique binding pose.

Conformational sampling, though extensive, cannot exhaustively explore all possible aptamer folds, leaving the possibility that some relevant binding modes were missed. The computational cost of docking large ensembles limits throughput. Therefore, parallelly, pool of aptamer conformers and the types of binding interactions can be used to train machine learning models, to accelerate generation of novel sequences, predictive modeling of binding affinities, and a deeper structure–activity relationship (SAR) understanding for nucleic acid ligands. However, the paucity of high-resolution experimental aptamer-protein structures hinders the validation of in silico predictions and the training of robust machine learning models. Nevertheless, the computational framework can reduce experimental load and provide mechanistic clarity, significantly shortening the time-to-deployment for aptamer-based biosensors and therapeutics, especially for rare or structurally ambiguous targets. Embedding computational methods into the aptamer discovery pipeline is no longer optional but imperative to realize the full potential of aptamer technology in precision diagnostics. We believe that the ease of these combined experimental and in silico molecular dynamic simulation workflows in the succession of an effective SELEX process may inspire the further mass development of aptamer-based sensors for molecular diagnostics including electronic or optical sensing platforms. Future device optimization before translation toward routine or point-of-care applications also need to evaluate long-term sensor stability. For this the aptamer-modified sensors also can be checked for storage-stability, long-term signal drift or reusability over days, weeks or months.

## Supplementary Information

Below is the link to the electronic supplementary material.

Supplementary Material 1

## Data Availability

The datasets (SELEX libraries sequencing data) generated and/or analysed during the current study are available in the DDBJ (DNA Data Bank of Japan) repository, Accession number: PRJDB40306 (https://ddbj.nig.ac.jp/search). The datasets (predicted macromolecular structures, PDB files) generated and/or analysed during the current study are available at the Mendeley Data repository under https://data.mendeley.com/datasets/gmr7n4d43s/1.
