# Supplementary material for: Single-sequence based gFET-aptasensors for the discrimination of apo- and holo-RBP4 in human serum
Source: Sci Rep. 2026 Jul 16;16:22287. doi: 10.1038/s41598-026-62460-z (PMC13373237; doi:10.1038/s41598-026-62460-z)
Supplement: Supplementary file 1 — Supplementary Material 1 [file 41598_2026_62460_MOESM1_ESM.docx]

**Supplementary Materials:**


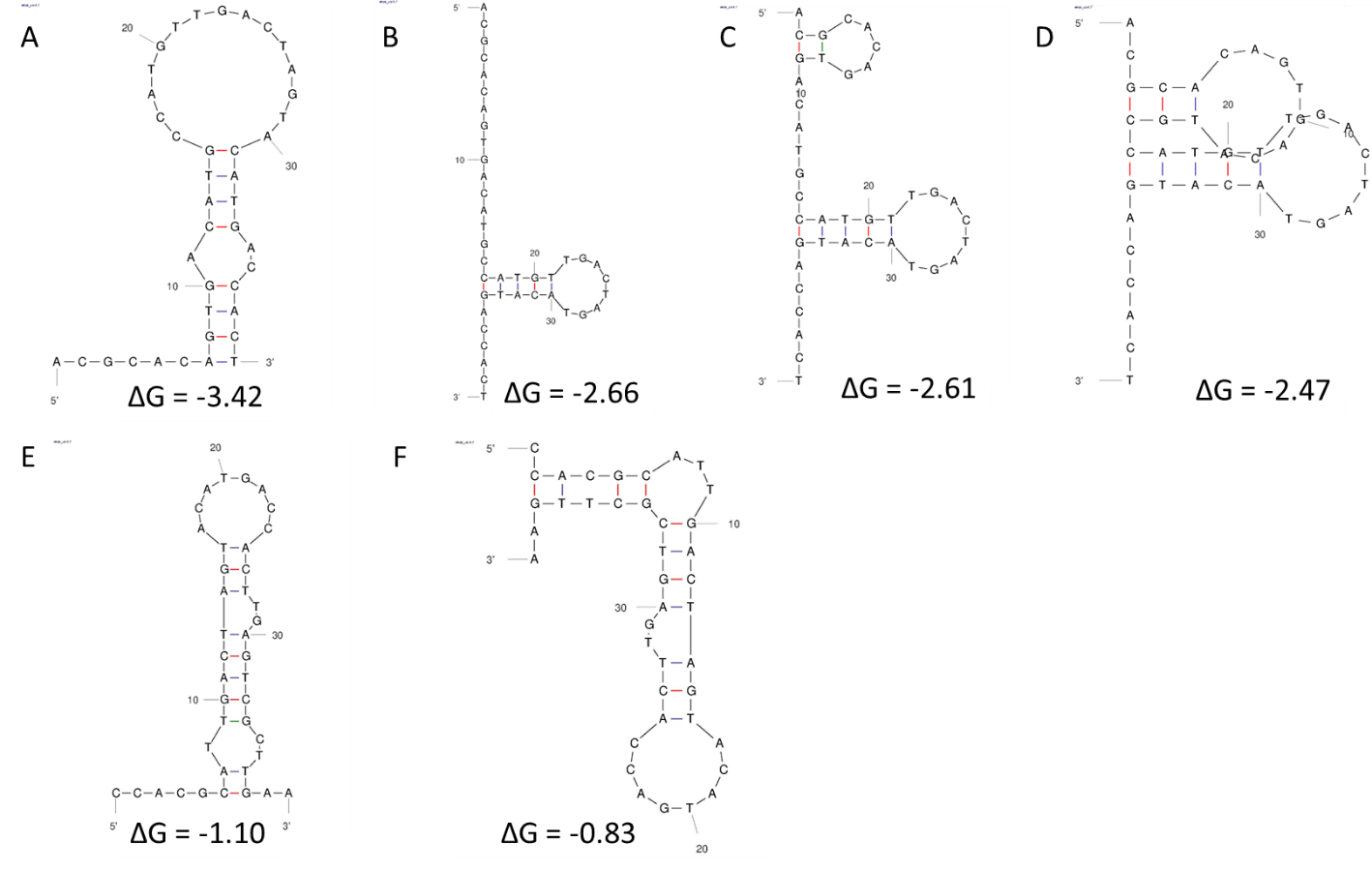


**Figure S1.** Predicted secondary structures and associated minimum free energy (ΔG) values of the active regions of aptamers A–D (apo-specific) and E–F (holo-specific), generated using Mfold v4.7. For modeling purposes, only the core binding regions (excluding primer-binding sites) were used: 5’-ACGCACAGTGACATGCCATGTTGACTAGTACATGACCACT-3’ for the apo-specific aptamer and 5’-CCACGCATTGACTAGTACATGACCACTTGAGTCGCTTGAA-3’ for the holo-specific aptamer.


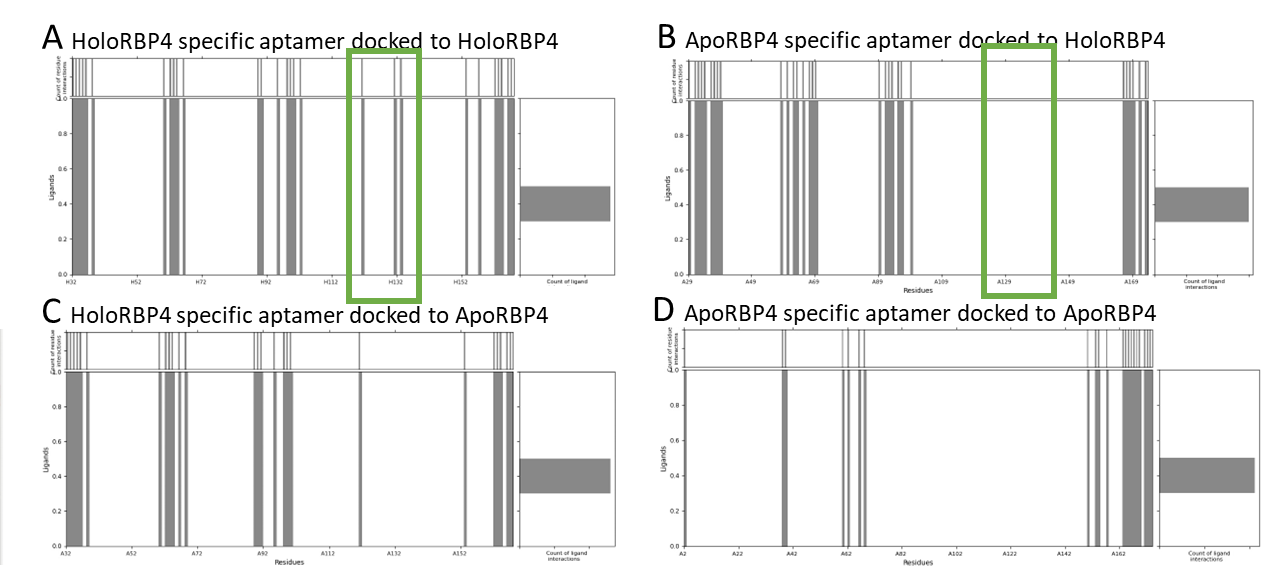


**Figure S2**. Residue-wise interaction fingerprint profiles of top-performing aptamer–RBP4 complexes. Post docking, structural interaction fingerprints (SIFts) were generated in the BioLuminate module in Schrödinger to quantify the nature and frequency of contacts between aptamer conformers and RBP4 protein forms (apo and holo). The plots represent normalized interaction frequencies across residues for all contact types, including hydrogen bonding, hydrophobic contacts, aromatic stacking, and electrostatic interactions. Each panel corresponds to a specific aptamer–protein complex: A and C show holo-specific aptamer with holo- and apo-RBP4 respectively; top-right and bottom-right represent holo-specific aptamer with holo- and apo-RBP4 respectively. The vertical bars on the right summarize the cumulative number of contacts each residue forms with the aptamer, highlighting key binding hotspots in the complex. The green box highlights few of the many differences in interactions that can be potentially mapped in the complexes: the presence and absence of interactions with residues ARG 121, ASP131 and TYR 133 when holo and apo aptamers were docked to holo-RBP4.

**
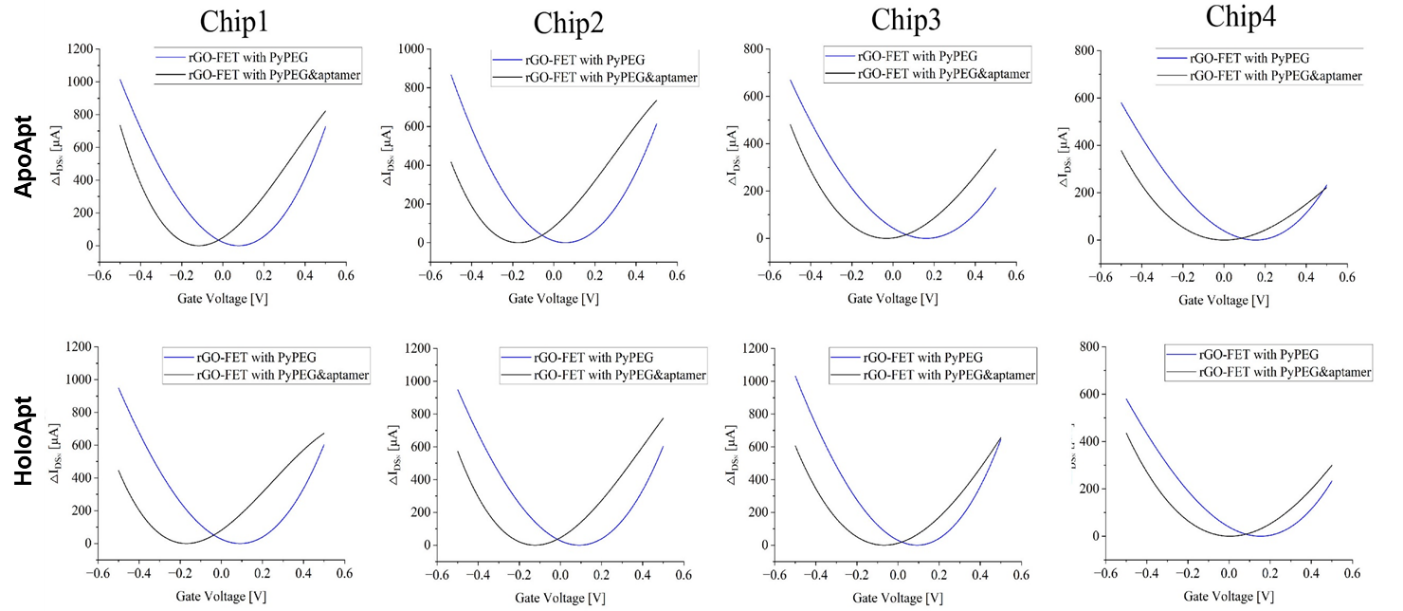
**

**Figure S3.** Layer-by-layer functionalization of reduced graphene surfaces using PyPEG linkers and subsequently EDC/NHS-mediated coupling of NH_2_-labeled ApoApt and HoloApt aptamers for each chip used for the experiments shown in Figures 4 and 5.


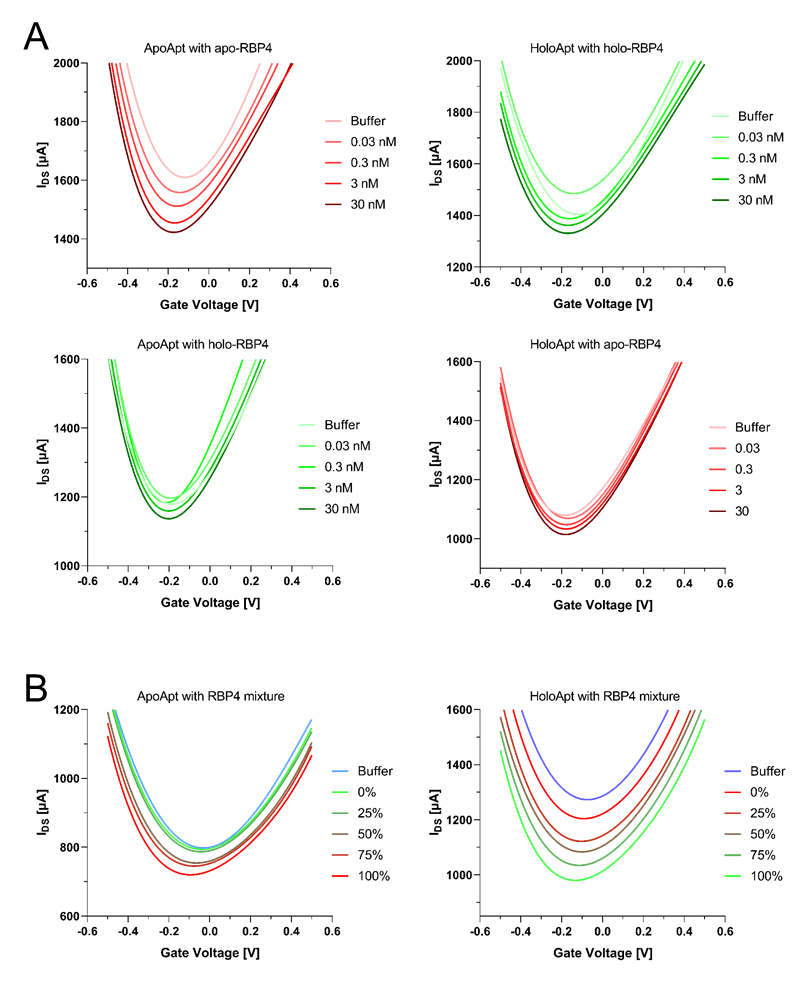


**Figure S4.** I_DS_V_G_ characterization of sensing devices obtained using sweeping the gate voltage from -0.5 V to 0.5 V. Binding of ApoApt and HoloApt aptamers to A) apo- or holo-RBP4 ranging from 0.03 – 30 nM and B) in defined mixtures with increasing amounts (0 – 100%) of apo- (red) and holo-RBP4 (green). I_DS_V_G_ curves represent mean values of experiments conducted in triplicate.


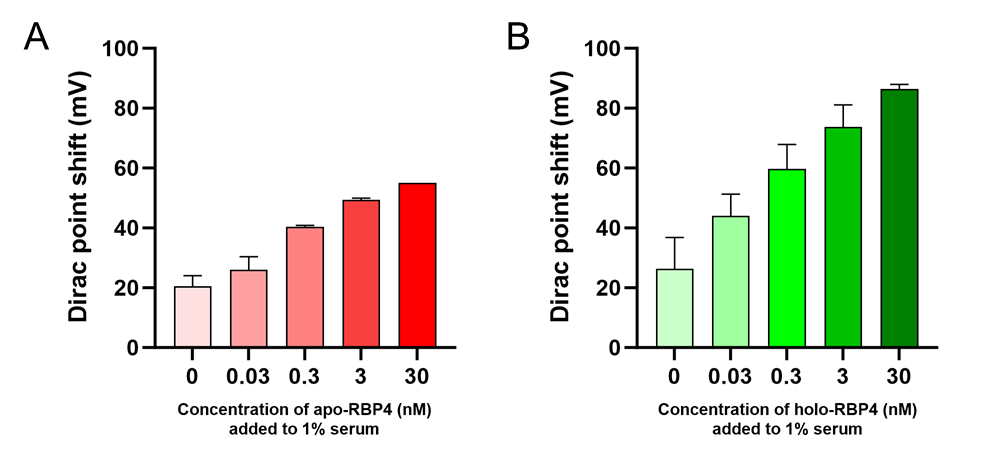


**Figure S5.** Dirac point shifts of A) ApoApt sensing of apo-RBP4 concentrations and B) HoloApt sensing of holo-RBP4 in 1% human serum from Figure 6.
